# Network-enabled drug repurposing identifies lithium to promote cardiomyocyte proliferation

**DOI:** 10.64898/2026.10.15.759417

**Published:** 2026-10-16

**Authors:** Kaitlyn L. Wintruba, Sunil Verma, Irfan S. Kathiriya, Aleksandra Kostina, Aitor Aguirre, Muge Kuyumcu-Martinez, Jeffrey J. Saucerman

## Abstract

The adult mammalian heart has minimal regenerative capacity, limited in part by endogenous cardiomyocyte (CM) renewal. Although CM proliferation is a promising target in both congenital and adult heart disease, no current therapies specifically target this process. Progress has been hindered by the complexity of CM proliferation signaling networks, which may require systems-level approaches. The overall goal of this study was to leverage systems modeling to identify FDA approved drugs that have the potential to be repurposed towards CM proliferation. To achieve this goal, we developed a computational drug repurposing framework that integrates drug-target interaction data with a previously developed logic-based network model of CM proliferation signaling. Using this framework, we identified 81 drugs that directly target nodes within the CM proliferation network, corresponding to 32 unique drug-target interactions. Virtual screening predicted 5 unique drug-target interactions that increase CM proliferation and 14 that decrease CM proliferation outputs. Lithium, a GSK3β inhibitor widely prescribed for bipolar disorder, was predicted and experimentally validated to promote CM proliferation through a Cyclin D/CDK4 pathway. *In vivo* , treatment of pregnant mice with lithium increased DNA synthesis in the CMs of their embryos. A retrospective analysis of the FDA Adverse Events Reporting System confirmed that relative to other mood stabilizers, *in utero* exposure to lithium was associated with an increased incidence of congenital heart defects, in males but not females. Together, this work provides a computational-experimental framework for repurposing FDA-approved drugs to modulate CM proliferation, which highlights therapeutic potential of lithium for adult CM regeneration but congenital risks to developing embryos.

